# Analysis of root canal anatomy configuration in permanent anterior teeth among the Pakistani subpopulations using cone-beam CT

**DOI:** 10.3389/froh.2026.1750120

**Published:** 2026-02-05

**Authors:** Mohmed Isaqali Karobari, Abdul Habeeb Adil, Azhar Iqbal, Hamza Arshad, Fatima Zahra, Fahad Umer, Irfan Maqbool, Venkata Suresh Venkataiah, Tahir Yusuf Noorani

**Affiliations:** 1 Department of Conservative Dentistry and Endodontics, Saveetha Dental College and Hospital, Saveetha Institute of Medical and Technical Sciences, Saveetha University, Chennai, Tamil Nadu, India; 2 Department of Dental Research, Saveetha Medical College, and Hospitals, Saveetha Institute of Medical and Technical Sciences, Saveetha University, Chennai, Tamil Nadu, India; 3 Department of Restorative Dentistry, College of Dentistry, Jouf University, Sakaka, Saudi Arabia; 4 Prosthodontics, Dental Section, Department of Surgery, The Aga Khan University, Karachi, Pakistan; 5 Operative Dentistry and Endodontics, Dental Section, Department of Surgery, The Aga Khan University, Karachi, Pakistan; 6 Private Dental Practitioner, Islamabad, Pakistan; 7 Clinical Sciences Department, Centre of Medical and Bio-Allied Health Science Research, College of Dentistry, Ajman University, Ajman, United Arab Emirates; 8 Conservative Dentistry Unit, School of Dental Sciences, Universiti Sains Malaysia, Kota Bharu 16150, Kelantan, Malaysia

**Keywords:** anterior teeth, CBCT, morphology, Pakistani subpopulation, prevalence, root canal

## Abstract

**Aim:**

This study aimed to analyse root canal configurations in permanent maxillary and mandibular anterior teeth using cone-beam CT among a Pakistani subpopulation.

**Method:**

A total of 7,653 maxillary and mandibular anterior teeth were included from the retrospective analysis of 707 CBCT scans from two institutions. The CBCT images were visualized using GALAXIS integrated within the Sirona Dental System. The scanning parameters were standardized at 85 kV, 7 mA, with a 15-second exposure time and a voxel size of 0.16 mm. Statistical analyses were performed using SPSS version 26, employing the Chi-square test with a significance level set at *p* < 0.05.

**Results:**

The predominant root canal configuration observed across all maxillary and mandibular anterior teeth was single root with single canal (^1^TN^1)^, comprising 84.66% of cases. Significant variations were noted in maxillary lateral incisors and mandibular canines across different age groups (*p* < 0.05), indicating age-related differences in root canal morphology in these teeth. Gender-based analysis revealed significant differences in root canal configurations for maxillary canines and mandibular central and lateral incisors (*p* < 0.05), with females exhibiting a higher prevalence of specific variants compared to males.

**Conclusion:**

The study successfully identified the predominant root canal configurations in a Pakistani subpopulation, revealing significant age and gender-related differences. The predominance of the single root with single (^1^TN^1^) canal configuration underscores its clinical relevance and prevalence across different demographic factors. The findings emphasize the importance of demographic considerations in endodontic treatment planning, contributing valuable regional data to the field.

## Introduction

Understanding the root canal morphology of anterior teeth is crucial for successful endodontic treatment (1). Variations in root canal anatomy can significantly influence the diagnosis, treatment planning, and prognosis of endodontic therapy (2). Numerous studies have indicated that root canal morphology varies significantly across different populations and ethnic groups, underscoring the need for population-specific data to enhance clinical outcomes (3–6).

The morphology of root canals in anterior teeth, which includes the central incisors, lateral incisors, and canines, is generally less complex than that of posterior teeth (7). However, variations still exist, such as differences in the number of canals, the presence of accessory canals, and the complexity of the canal system (8). These variations can pose challenges during endodontic procedures, potentially leading to incomplete debridement, missed canals, and treatment failures if not properly understood and managed (9).

In Pakistan, the population is characterized by diverse ethnic and genetic backgrounds, which may contribute to variations in dental anatomy (10). Previous studies on root canal morphology have predominantly focused on Western and East Asian populations, with limited data available for South Asian demographics, including Pakistan (4, 11, 12). This lack of region-specific information can result in less effective endodontic treatment for Pakistani patients due to a reliance on generalized data that may not accurately reflect their unique anatomical features (13).

Several studies have highlighted the importance of understanding root canal morphology for effective endodontic treatment (14–18). Vertucci (9) provided a seminal classification of root canal systems, identifying eight different types based on their configuration (9). Subsequent research has expanded on Vertucci's work, demonstrating significant variation among different ethnic groups. A study by Cleghorn et al. (19) showed notable differences in root canal morphology between Caucasian and Asian populations (19).

In the context of the Pakistani population, few studies have explored root canal morphology in detail. A study by Mir et al. (13) used CBCT to examine the root canal anatomy of mandibular incisors in a sample of Pakistani patients, revealing a higher prevalence of two canals (26.2%) compared to previous studies on other populations (13). Further, research conducted by Prena Moorpani et al. (20) established the occurrence rate of the second canal in mandibular lateral incisors among the Pakistani subpopulations. The researchers determined that mandibular lateral incisors were more commonly found to have Type I canal morphology, while incisors with two canals were most frequently found to have Type III design (20). However, comprehensive data on the root canal morphology of all anterior teeth in various Pakistani subpopulations remains sparse.

The Ahmed et al. (21) classification system was chosen for this study due to its comprehensive approach in categorizing root canal morphology, offering a detailed framework that includes variations based on the number of canals, pathways, and additional anatomical features (21). While Vertucci's classification has been widely used, it is limited in capturing the full complexity of root canal systems and is based on older methodologies that may overlook more intricate variations (22). Ahmed et al.'s system, by incorporating advancements in imaging technology like CBCT, provides a more precise and nuanced understanding of root canal anatomy, making it particularly useful for identifying fewer common configurations and enhancing the accuracy of endodontic treatments (21). Although CBCT-based investigations of anterior tooth morphology have been reported from several neighbouring and ethnically comparable populations, the available literature remains limited in terms of sample size, analytical depth, and classification approach. Most previous studies from South Asia and surrounding regions have primarily focused on reporting prevalence patterns using traditional classification systems, which may underestimate the complexity and rarity of canal configurations.

This study aimed to provide valuable insights into the prevalence of various canal configurations and anatomical variations in permanent maxillary and mandibular anterior teeth, as observed using CBCT, among a Pakistani subpopulation. Such information is essential for dental practitioners to tailor their endodontic approaches to the specific needs of this population, ultimately enhancing treatment efficacy and patient outcomes.

## Methodology

### Ethical consideration

This retrospective study was conducted in accordance with the principles of the Declaration of Helsinki and received ethical approval from the Ethical Review Committee of Aga Khan University, Karachi, Pakistan (Approval No: 2024-1008-29149). All CBCT scans used in this study were originally obtained for diagnostic and therapeutic purposes unrelated to the present research. Patient confidentiality was strictly maintained, and all data were fully anonymised prior to analysis. Informed consent was obtained from all participants and/or their legal guardians at the time of imaging in accordance with institutional protocols.

### Sample selection

The study involved a retrospective analysis of 707 CBCT scans from two institutions: 592 scans were provided by the Aga Khan University dental clinics in Karachi, and 115 scans were obtained from Jinnah MRI and Body Scan CBCT in Lahore. The selection of scans was carried out using a simple random sampling technique.

### Inclusion criteria

Patients with fully erupted permanent anterior teeth (central incisors, lateral incisors, and canines) were included. Teeth must not have undergone previous endodontic therapy. Only high-resolution CBCT scans with clear visualisation of the root canal systems were included.

### Exclusion criteria

For this retrospective study, patients with evidence of orthodontic treatment, dental implants, crowns, bridges, or extensive restorations would have been excluded by reviewing their clinical records and identifying these features on the CBCT scans. Additionally, teeth with severe caries, fractures, or other pathological conditions would have been excluded based on radiographic findings visible in the CBCT images, ensuring that the root canal morphology could be accurately assessed without interference from these factors.

### Analysis of root canal morphology

The CBCT images from Karachi were analysed using GALAXIS version 1.9 (SICAT GmbH and Co. KG, Bonn, Germany) on a 21-inch monitor. These scans were obtained using a Sirona device (Bensheim, Germany) at 85 kV and 7 mA, with a voxel size of 0.16 mm and an exposure time of 15 s. For the Lahore scans, the Planmeca Promax 3D Classic (Planmeca, Helsinki, Finland) was used, and images were viewed with Planmeca Romexis version 6.4.3.33 on a 21-inch monitor. These scans were conducted at 90 kV and 10 mA, with a voxel size of 0.2 mm and an exposure time of 15 s. An expert endodontist and two dental residents evaluated each tooth in coronal, axial, and sagittal sections using the classification system proposed by Ahmed et al. (21). Data on each tooth's gender, age, and classification were recorded in an Excel spreadsheet.

Although CBCT scans were obtained from two different centres using distinct devices and acquisition parameters, both protocols employed voxel sizes (0.16 mm and 0.2 mm) that are widely accepted for reliable visualization of root canal morphology. To ensure consistency of interpretation across datasets, all images were assessed under standardized viewing conditions by a calibrated evaluation team.

### Calibration

Before initiating the study, a standardized approach was established using a sample of sixty CBCT images. Two dental residents and an endodontist conducted the calibration process together. Images were evaluated in axial, sagittal, and coronal sections to ensure accurate classification of root canal morphology using the Ahmed et al. (21) system. The calibration took place in a controlled environment: a dimly lit room to minimize glare, and all images were viewed on a medical-grade monitor (Dell Ultrasharp U2718Q, 27-inch, 4 K resolution) to ensure optimal image quality and consistency across reviews. Each examiner received training in a series of workshops led by an experienced endodontist. These sessions focused on identifying and interpreting the root canal systems in CBCT scans, following the Ahmed et al. classification. The scoring system was based on consensus and applied consistently to root canal morphology. The repeatability assessment involved the same observer re-analysing the same CBCT images at two different intervals, separated by two weeks. The reproducibility assessment was done by comparing the evaluations of different observers, where each examiner independently analysed 60 scans. Discrepancies in interpretations were reviewed jointly, and a consensus was reached after group discussions. Initially, a 10% disagreement rate was observed, which significantly reduced with experience and further training. The calibration achieved a Cohen's kappa coefficient of 0.8, indicating strong agreement among the dental residents and the endodontist, demonstrating reliable intra- and inter-examiner consistency in image interpretation.

### Statistical analysis

Statistical analysis was performed using SPSS version 26 (SPSS Inc., Chicago, Illinois, USA). Intra- and inter-examiner reliability was evaluated using the Cohen kappa coefficient to ensure consistency in the assessments. The chi-square test was employed to assess the prevalence of different root canal morphologies across various age groups and genders, with a significance level set at *p* < 0.05.

## Results

The study comprehensively analysed the root canal configurations of 7,653 maxillary and mandibular anterior teeth, providing insights into their distribution across different tooth types and demographic factors.

The demographic characteristics of the study population are summarized in Table 1. The age distribution showed that the most prominent groups were 21–30 years (22.2%) and 31–40 years (22.5%), with a mean age of 38.11 ± 14.98. Gender distribution was nearly balanced, with 47.2% males and 52.8% females.

**Table 1:** Descriptive statistics of demographic characteristics (*n* = 707).

Variables	Frequency (*n*)	Percentage (%)
Age		
10–20	88	12.4
21–30	157	22.2
31–40	159	22.5
41–50	134	19.0
51–60	106	15.0
61–70	58	8.2
71–80	5	0.7
Gender		
Male	334	47.2
Female	373	52.8

The distribution of different root canal configurations among various tooth types is detailed in Table 2. Across all teeth examined, the ^1^TN^1^ configuration emerged as the predominant pattern, comprising 84.66% of the total cases. Specifically, this configuration was most prevalent in maxillary central incisors (*n* = 1,211 teeth), maxillary lateral incisors (*n* = 1,205 teeth), maxillary canines (*n* = 1,133 teeth), mandibular central incisors (*n* = 880 teeth), mandibular lateral incisors (*n* = 871 teeth), and mandibular canines (*n* = 1,179 teeth). In addition to the predominant ^1^TN^1^ pattern, the study identified other less frequent configurations. The ^1^TN^1-2-1^ configuration, for instance, accounted for 12.27% of cases, primarily seen in mandibular central incisors (392 teeth) and mandibular lateral incisors (433 teeth). Minor variants such as ^1^TN^1-2^ (1.64%) and ^1^TN^2-1^ (0.62%) were observed sporadically across different tooth types. Rare configurations included ^2^TN^1^B^1^L^1^ (0.50%), ^1^TN^1-2-1-2^ (0.10%), ^1^TN^2-1-2-1^ (0.09%), and exceedingly rare configurations like ^1^TN^1-3^ (0.03%), ^1^TN^2-1-2^ (0.01%), and ^1^TN^2-1-2-1-2-1^ (0.01%).

**Table 2:** Distribution of root canal configuration of maxillary and mandibular anterior teeth.

Classification	Maxillary central incisors	Maxillary lateral incisors	Maxillary canines	Mandibular central incisors	Mandibular lateral incisors	Mandibular canines	Overall *n*(%)
^1^TN^1^	1,211	1,205	1,133	880	871	1,179	6,479 (84.66)
^1^TN^2-1^	11	7	9	7	9	4	47 (0.62)
^1^TN^1-2-1^	4	1	11	392	433	98	939 (12.27)
^1^TN^1-2^	14	22	49	10	10	20	125 (1.64)
^1^TN^2-1-2^	0	0	0	0	1	0	1 (0.01)
^1^TN^1-2-1-2^	0	0	0	4	4	0	8 (0.10)
^1^TN^2-1-2-1^	0	0	0	5	1	1	7 (0.09)
^2^TN B^1^L^1^	0	0	0	0	1	37	38 (0.50)
^1^TN^1-2-1-2-1^	0	0	0	2	3	0	5 (0.06)
^1^TN^1-3^	0	2	1	0	0	0	3 (0.03)
^1^TN^2-1-2-1-2-1^	0	0	0	1	0	0	1 (0.01)
Total	1,240	1,237	1,203	1,301	1,333	1,339	7,653 (100)

### Maxillary anteriors

**Figure 1:**
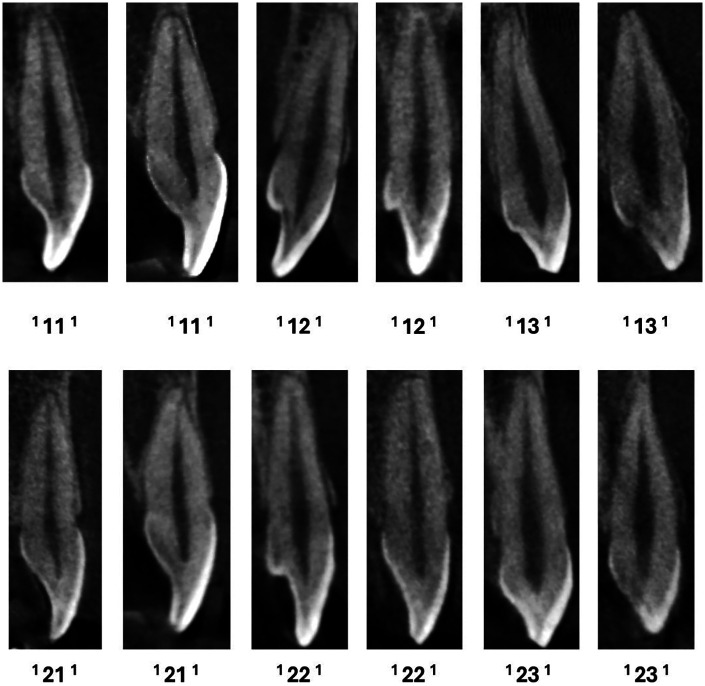
CBCT sagittal view of maxillary anteriors with a single canal.

**Figure 2:**
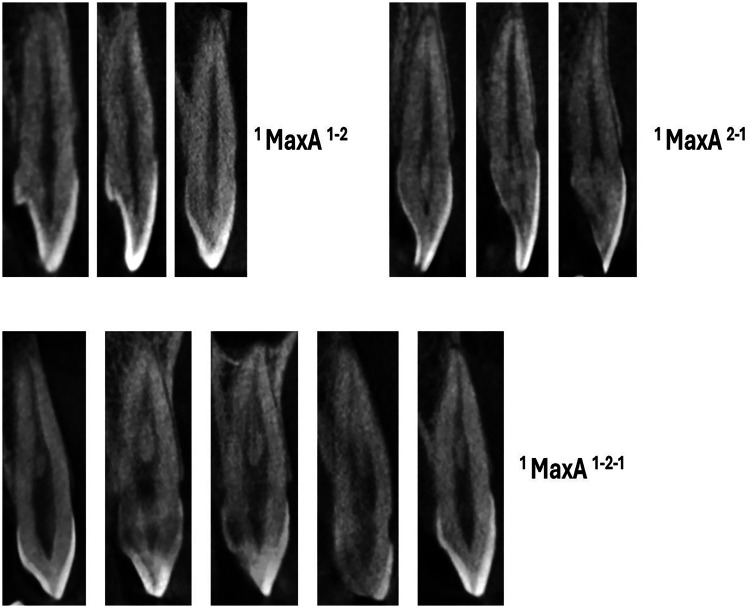
CBCT sagittal view of maxillary anteriors with two and three code classifications (maxA-maxillary anteriors).

Further analysis focused on age-related variations in root canal configurations among maxillary anterior teeth, as detailed in Table 3. The predominant ^1^TN^1^ configuration was consistently found across different age groups, indicating stability in root canal morphology over time. Variants such as ^1^TN^2-1^ and ^1^TN^1-2-1^ were observed sporadically, with no statistically significant differences across age groups noted for maxillary central incisors and canines (*p* > 0.05). In contrast, significant age-related differences were observed in root canal configurations for maxillary lateral incisors (*p* < 0.05) (Figures 1, 2).

**Table 3:** Distribution of root canal configuration of maxillary anterior teeth concerning age.

Teeth	Age Group	Variants					Total	Chi-square value	*p*-value
		^1^TN^1^	^1^TN^2-1^	^1^TN^1-2-1^	^1^TN^1-2^	^1^TN^1-3^			
		*n* (%)	*n* (%)	*n* (%)	*n* (%)	*n* (%)	*n* (%)		
Maxillary Central Incisors	10–20	153 (98.08)	3 (1.92)	0	0	0	156 (100)	25.011	0.125
	21–30	216 (96.87)	3 (1.34)	3 (1.34)	1 (0.45)	0	223 (100)		
	31–40	268 (97.46)	3 (1.09)	0	4 (1.45)	0	275 (100)		
	41–50	253 (97.69)	2 (0.77)	0	4 (1.54)	0	259 (100)		
	51–60	196 (99.00)	0	1 (0.50)	1 (0.50)	0	198 (100)		
	61–70	115 (96.64)	0	0	4 (3.36)	0	119 (100)		
	71–80	10 (100)	0	0	0	0	10 (100)		
Maxillary Lateral Incisors	10–20	154 (98.72)	0	1 (0.64)	0	1 (0.64)	156 (100)	41.978	**0.013**
	21–30	227 (96.60)	5 (2.12)	0	3 (1.28)	0	235 (100)		
	31–40	266 (98.16)	0	0	4 (1.48)	1 (0.36)	271 (100)		
	41–50	245 (96.08)	2 (0.79)	0	8 (3.13)	0	255 (100)		
	51–60	197 (99.50)	0	0	1 (0.50)	0	198 (100)		
	61–70	106 (94.65)	0	0	6 (5.35)	0	112 (100)		
	71–80	10 (100)	0	0	0	0	10 (100)		
Maxillary Canines	10–20	124 (96.88)	0	0	4 (3.12)	0	128 (100)	29.685	0.195
	21–30	217 (93.94)	4 (1.73)	1 (0.43)	9 (3.90)	0	231 (100)		
	31–40	254 (93.74)	3 (1.10)	5 (1.84)	9 (3.32)	0	271 (100)		
	41–50	238 (92.24)	1 (0.39)	2 (0.79)	16 (6.20)	1 (0.38)	258 (100)		
	51–60	186 (96.38)	1 (0.52)	3 (1.55)	3 (1.55)	0	193 (100)		
	61–70	106 (94.65)	0	0	6 (5.35)	0	112 (100)		
	71–80	8 (80.0)	0	0	2 (20.0)	0	10 (100)		

Significant value <0.05; Chi-square test.

Bold value indicates statistical significance.

Gender-based analysis (Table 4) revealed notable patterns in root canal configurations among maxillary anterior teeth. Both males (97.58%) and females (97.74%) predominantly exhibited the ^1^TN^1^ configuration in maxillary central incisors, with minimal differences noted in other variants such as ^1^TN^2-1^ and ^1^TN^1-2-1^. Statistical analysis showed no significant gender differences in root canal configurations for maxillary central and lateral incisors (*p* > 0.05). In contrast, significant gender-related differences were observed in maxillary canines (*p* < 0.05), with females showing a higher prevalence of ^1^TN^1-2-1^ and ^1^TN^1-3^ variants compared to males.

**Table 4:** Distribution of root canal configuration of maxillary anterior teeth concerning gender.[24]

Teeth	Gender	Variants					Total	Chi-square value	*p*-value
		^1^TN^1^	^1^TN^2-1^	^1^TN^1-2-1^	^1^TN^1-2^	^1^TN^1-3^			
		*n* (%)	*n* (%)	*n* (%)	*n* (%)	*n* (%)	*n* (%)		
Maxillary Central Incisors	Male	563 (97.58)	6 (1.04)	2 (0.34)	6 (1.04)	0	577 (100)	0.380	0.944
	Female	648 (97.74)	5 (0.76)	2 (0.30)	8 (1.20)	0	663 (100)		
Maxillary Lateral Incisors	Male	565 (97.76)	2 (0.34)	0	11 (1.90)	0	578 (100)	3.666	0.453
	Female	640 (97.12)	5 (0.76)	1 (0.15)	11 (1.67)	2 (0.30)	659 (100)		
Maxillary Canines	Male	561 (95.41)	7 (1.19)	6 (1.02)	14 (2.38)	0	588 (100)	12.376	**0** **.** **015**
	Female	572 (93.00)	2 (0.32)	5 (0.82)	35 (5.70)	1 (0.16)	615 (100)		

Significant value <0.05; Chi-square test.

Bold value indicates statistical significance.

### Mandibular anteriors

**Figure 3:**
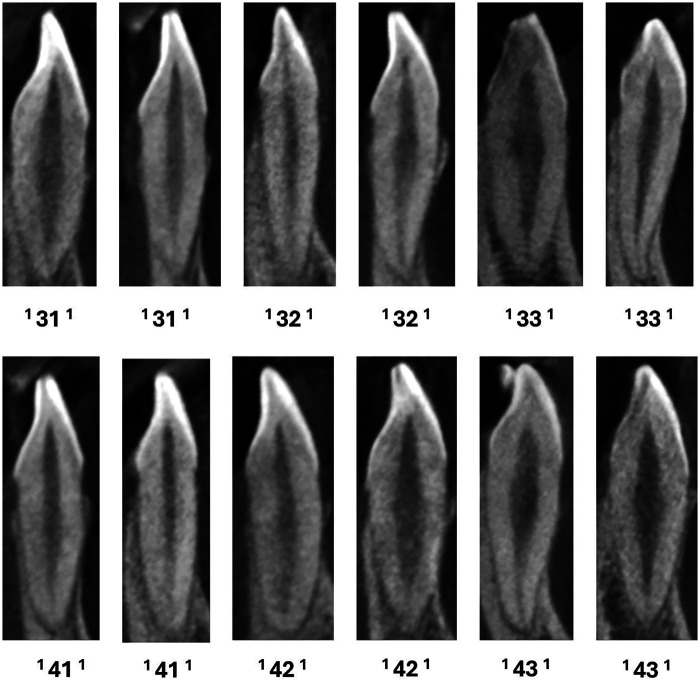
CBCT sagittal view of mandibular anteriors with a single canal.

**Figure 4:**
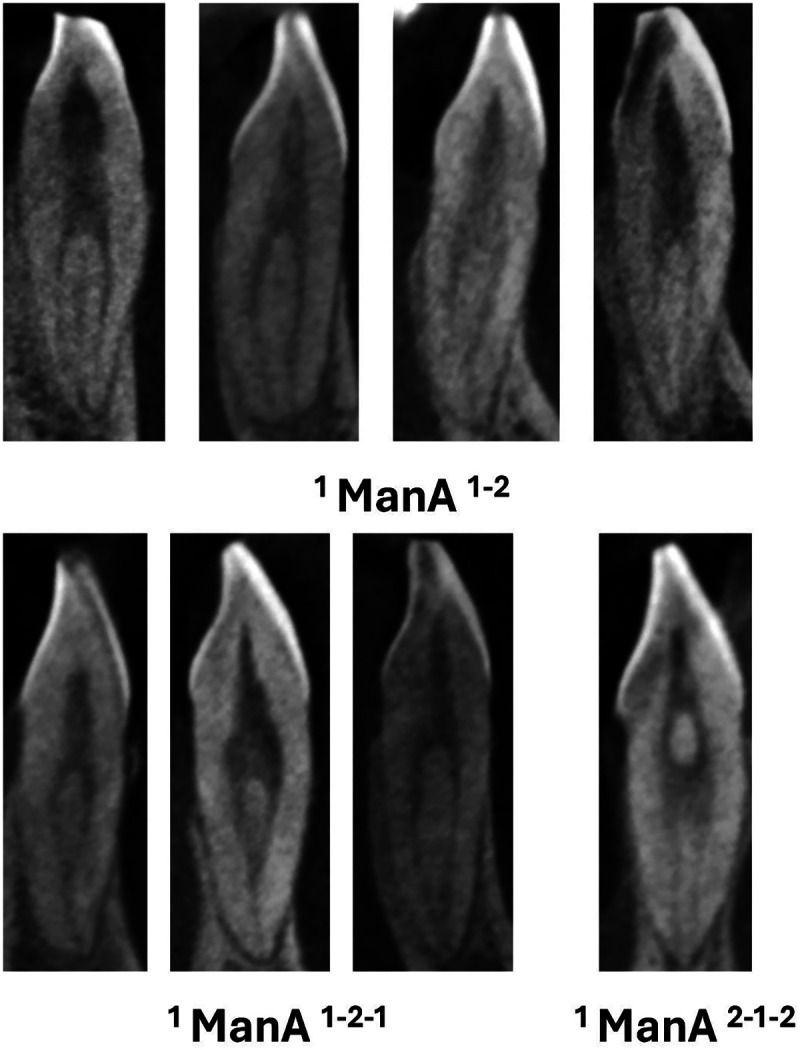
CBCT sagittal view of mandibular anteriors with two and three code classifications (manA-mandibular anteriors).

**Figure 5:**
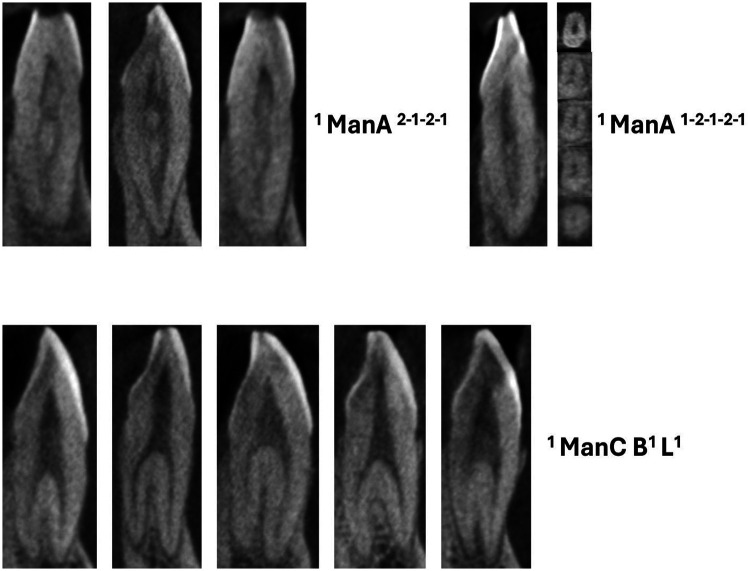
CBCT sagittal view of mandibular anteriors with more than three code classifications (manA-mandibular anteriors) and mandibular canines with two roots (manC-mandibular canines).

Turning to mandibular anterior teeth (Table 5), similar trends were observed with the predominance of the ^1^TN^1^ configuration across all age groups. The ^1^TN^1-2-1^ configuration, while less frequent, also appeared consistently across different age brackets. Unlike maxillary teeth, significant age-related differences in root canal configurations were noted for mandibular canines (*p* < 0.05) (Figures 3–5).

**Table 5:** Distribution of root canal configuration of mandibular anterior teeth concerning age.

Teeth	Age Group	Variants										Total	Chi-square value	*p*-value
		^1^TN^1^	^1^TN^2-1^	^1^TN^1-2-1^	^1^TN^1-2^	^1^TN^2-1-2^	^1^TN^1-2-1-2^	^1^TN^2-1-2-1^	^2^TN B^1^L^1^	^1^TN^1-2-1-2-1^	^1^TN^2-1-2-1-2-1^			
		*n* (%)	*n* (%)	*n* (%)	*n* (%)	*n* (%)						*n* (%)		
Mandibular Central Incisors	10–20	107 (61.50)	0	64 (36.78)	2 (1.14)	0	1 (0.58)	0	0	0	0	174 (100)	55.670	0.077
	21–30	181 (71.55)	4 (1.58)	62 (24.51)	4 (1.58)	0	1 (0.39)	0	0	0	1 (0.39)	253 (100)		
	31–40	179 (63.70)	3 (1.06)	95 (33.81)	2 (0.71)	0	1 (0.36)	1 (0.36)	0	0	0	281 (100)		
	41–50	173 (66.54)	0	81 (31.15)	1 (0.38)	0	1 (0.38)	4 (1.55)	0	0	0	260 (100)		
	51–60	144 (72.37)	0	53 (26.63)	0	0	0	0	0	2	0	199 (100)		
	61–70	90 (72.59)	0	33 (26.61)	1	0	0	0	0	0	0	124 (100)		
	71–80	6 (60.0)	0	4 (40.0)	0	0	0	0	0	0	0	10 (100)		
Mandibular Lateral Incisors	10–20	114 (65.14)	0	58 (33.14)	2 (1.14)	0	1 (0.58)	0	0	0	0	175 (100)	39.096	0.817
	21–30	175 (68.90)	2 (0.79)	70 (27.55)	4 (1.59)	1 (0.39)	1 (0.39)	0	0	1 (0.39)	0	254 (100)		
	31–40	171 (60.64)	5 (26.60)	102 36.17)	1 (0.35)	0	1 (0.35)	1 (0.35)	0	1 (0.35)	0	282 (100)		
	41–50	167 (61.18)	1 (0.36)	102 (37.38)	1 (0.36)	0	1 (0.36)	0	1 (0.36)	0	0	273 (100)		
	51–60	142 (67.95)	1 (0.47)	64 (30.63)	1 (0.47)	0	0	0	0	1 (0.47)	0	209 (100)		
	61–70	96 (73.85)	0	33 (25.39)	1 (0.76)	0	0	0	0	0	0	130 (100)		
	71–80	6 (60.0)	0	4 (40.0)	0	0	0	0	0	0	0	10 (100)		
Mandibular Canines	10–20	154 (93.34)	0	6 (3.63)	2 (1.21)	0	0	0	3 (1.82)	0	0	165 (100)	44.963	**0** **.** **039**
	21–30	222 (89.52)	3 (1.20)	6 (2.42)	4 (1.62)	0	0	1 (0.40)	12 (4.84)	0	0	248 (100)		
	31–40	240 (85.10)	1 (0.36)	30 (10.64)	3 (1.06)	0	0	0	8 (2.84)	0	0	282 (100)		
	41–50	235 (85.46)	0	30 (10.90)	3 (1.09)	0	0	0	7 (2.55)	0	0	275 (100)		
	51–60	200 (89.29)	0	14 (6.25)	5 (2.23)	0	0	0	5 (2.23)	0	0	224 (100)		
	61–70	117 (87.32)	0	12 (8.95)	3 (2.23)	0	0	0	2 (1.50)	0	0	134 (100)		
	71–80	11 (100)	0	0	0	0	0	0	0	0	0	11 (100)		

Significant value <0.05; Chi-square test.

Bold value indicates statistical significance.

For mandibular anterior teeth (Table 6), gender-based analysis demonstrated nuanced differences in root canal configurations. Mandibular central incisors exhibited a higher prevalence of the ^1^TN^1^ configuration in females (69.89%) compared to males (64.99%), with statistical significance noted (*p* < 0.05). Similarly, significant gender-related differences were observed in mandibular lateral incisors (*p* < 0.05), highlighting variations in root canal morphology between males (61.04%) and females (69.04%). Conversely, no significant gender differences were found in root canal configurations for mandibular canines (*p* > 0.05), indicating a consistent distribution of root canal patterns across genders in this tooth type.

**Table 6:** Distribution of root canal configuration of mandibular anterior teeth concerning gender.[28]

Teeth	Gender	Variants										Total	Chi-square value	*p*-value
		^1^TN^1^	^1^TN^2-1^	^1^TN^1-2-1^	^1^TN^1-2^	^1^TN^2-1-2^	^1^TN^1-2-1-2^	^1^TN^2-1-2-1^	^2^TN B^1^L^1^	^1^TN^1-2-1-2-1^	^1^TN^2-1-2-1-2-1^			
		*n* (%)	*n* (%)	*n* (%)	*n* (%)	*n* (%)						*n* (%)		
Mandibular Central Incisors	Male	388 (64.99)	6 (1.00)	192 (32.17)	3 (0.51)	0	1 (0.16)	5 (0.85)	0	1 (0.16)	1 (0.16)	597 (100)	15.933	**0** **.** **026**
	Female	492 (69.89)	1 (0.14)	200 (28.41)	7 (0.99)	0	3 (0.42)	0	0	1 (0.15)	0	704 (100)		
Mandibular Lateral Incisors	Male	376 (61.04)	3 (0.49)	231 (37.51)	2 (0.32)	0	0	1 (0.16)	1 (0.16)	2 (0.32)	0	616 (100)	22.611	**0** **.** **004**
	Female	495 (69.04)	6 (0.84)	202 (28.18)	8 (1.11)	1 (0.14)	4 (0.55)	0	0	1 (0.14)	0	717 (100)		
Mandibular Canines	Male	561 (89.62)	3 (0.48)	41 (6.55)	4 (0.64)	0	0	0	17 (2.71)	0	0	626 (100)	9.197	0.101
	Female	618 (86.68)	1 (0.14)	57 (7.99)	16 (2.24)	0	0	1 (0.14)	20 (2.81)	0	0	713 (100)		

Significant value <0.05; Chi-square test.

Bold value indicates statistical significance.

## Discussion

This study employed CBCT to investigate morphological variations in the root canal systems of 7,653 permanent maxillary and mandibular anterior teeth in a subset of Pakistani individuals, utilising a new classification system proposed by Ahmed et al. (21). The findings provide significant insights into the distribution patterns across different tooth types and demographic factors. Numerous studies have examined the morphology of maxillary and mandibular anterior teeth in various populations (1, 4, 11). To study tooth morphology, several techniques have been utilised, including demineralisation and (9, 23, 24), staining and grinding (25), and more recently, CBCT (12, 26, 27).

While several CBCT studies on anterior tooth morphology have been conducted in regional and ethnically related populations, the present investigation offers important extensions to the existing body of evidence. First, the large sample size of 7,653 teeth provides a more reliable and representative assessment of anterior root canal morphology than previously reported studies from this region. Second, the use of the Ahmed et al. (21) classification system allowed the detection of complex and rare canal configurations that are often underreported when traditional classification systems are employed. Third, this study demonstrates statistically significant age- and gender-related variations in specific tooth groups, offering novel demographic perspectives that have not been consistently documented in prior studies. These combined methodological and analytical strengths enhance the clinical applicability of the findings and provide new insights into population-specific endodontic anatomy.

CBCT offers a three-dimensional view of anatomical structures and is recognised as an effective tool for analysing root canal morphology (28, 29). Studies have demonstrated its efficacy in assessing the root canal system. CBCT has shown comparable accuracy to clearing and staining methods in detecting the number of root canals and has even surpassed the clearing technique (30, 31). As a straightforward, practical, non-invasive, and reliable method, CBCT is highly regarded for evaluating root canal morphology (32, 33).

The study findings underscore the predominance of the ^1^TN^1^ configuration, which accounted for 84.66% across all maxillary and mandibular anterior tooth types. The high prevalence of the ^1^TN^1^ configuration in this study corroborates findings from previous studies. For example, a study by Alhumaidi et al. (34) reported similar dominance of single canal configurations in maxillary and mandibular anterior teeth, emphasizing the anatomical consistency across populations (34). The present study's findings of 1,211 maxillary central incisors, 1,205 maxillary lateral incisors, and 1,133 maxillary canines with the ^1^TN^1^ configuration echo the results reported by Taha et al. (35), who also found a high frequency of single canal systems in maxillary anterior teeth (35).

Additionally, the study identified less frequent configurations, such as the ^1^TN^1-2-1^ configuration (12.27%), predominantly in mandibular central and lateral incisors. This finding is particularly noteworthy as it highlights a potential area of complexity in root canal treatments for these teeth. A study by Siddique et al. (36) supports this observation, noting a higher incidence of additional canals and more complex root canal systems in mandibular incisors compared to maxillary counterparts (36). The study's exploration of rare configurations, including ^2^TN B^1^L^1^ (0.50%), ^1^TN^1-2-1-2^ (0.10%), and ^1^TN^2-1-2-1^ (0.09%), adds to the understanding of anatomical variations in root canal systems. These findings are consistent with those of Iqbal et al. (37), who documented the presence of complex and varied root canal configurations in a minority of cases, stressing the importance of thorough radiographic examination and careful exploration during endodontic procedures to identify such variations (37).

### Maxillary anteriors

Age-related analysis in this study revealed that the ^1^TN^1^ configuration remained consistent across different age groups for maxillary central incisors and canines, with no significant differences. However, for maxillary lateral incisors, significant age-related differences in root canal configurations were observed. This stability in the predominant configuration suggests a consistent root canal morphology over time, except for the maxillary lateral incisors, which exhibited more variation. Comparison with other studies reveals both similarities and differences. For instance, Altunsoy et al. (4) and Jain et al. (38) also reported a high prevalence of the ^1^TN^1^ configuration in various populations, aligning with our findings. However, these studies did not highlight significant age-related differences in lateral incisors as observed in this study. Studies by Weng et al. (39) and Kuzekanni et al. (40) employed different methodologies, such as demineralization and staining, which corroborated the prevalence of the ^1^TN^1^ configuration but lacked the detailed age-specific analysis provided by CBCT. More recent research utilizing CBCT, such as by Chen et al. (41) and Karobari et al. (42), demonstrated similar findings regarding the accuracy and reliability of this imaging technique in identifying root canal configurations. However, our study's focus on a specific demographic subset adds valuable data on regional anatomical variations, which were not extensively covered in previous works. Thus, while the ^1^TN^1^ configuration appears to be a universally predominant pattern, regional and age-specific variations, especially in maxillary lateral incisors, highlight the importance of demographic considerations in endodontic studies.

The gender-based analysis of root canal configurations in maxillary anterior teeth reveals distinct patterns. Both male (97.58%) and female (97.74%) subjects predominantly exhibited the ^1^TN^1^ configuration in maxillary central incisors, with minimal differences in less common variants like ^1^TN^2-1^ and ^1^TN^1-2-1^. The statistical analysis confirms no significant gender differences in the root canal configurations of maxillary central and lateral incisors, aligning with the previous studies (43–45). However, significant gender-related differences were observed in maxillary canines. Females demonstrated a higher prevalence of the ^1^TN^1-2-1^ and ^1^TN^1-3^ variants compared to males. This variation agrees with studies like those by Nikkerdar et al. (46) and Naseri et al. (47), where significant gender differences were reported in the maxillary canines. These discrepancies might be attributed to genetic and environmental factors influencing root canal morphology in different populations.

### Mandibular anteriors

The analysis of mandibular anterior teeth reveals that the ^1^TN^1^ configuration is predominant across all age groups. These findings align with research by Zhengyan et al. (48), who reported similar configurations in mandibular anterior teeth. Significant age-related differences in root canal configurations were observed for mandibular canines. Additionally, the findings showed that the 21–30 age group had a higher prevalence (4.84%) of mandibular canines with two canals and more complicated root canal anatomy than the other age groups. This is consistent with the findings of Ahmed et al. (21), who documented that the incidence of two roots in the mandibular canines is as much as 15%. Similarly, Siddique et al. (36) reported the incidence of two roots of mandibular canines at 4.9% among Pakistani populations. The observed variations can be attributed to differences in racial background, genetic components, and methodologies employed in research. Contrary to our study's findings, previous studies (23, 44, 49, 50) have indicated a lower occurrence of multi-root canals in mandibular canines compared to central incisors and lateral incisors. Additionally, most of these studies have provided evidence supporting a higher prevalence of multi-root canals in mandibular lateral incisors. When compared to the other mandibular anterior teeth, the findings of this study revealed that the incidence of multi-root canals was significantly higher in the mandibular canines. Variances in examination procedures, classification methods, sample size, and regional and ethnic distributions of teeth may be responsible for the differences when compared to the findings of earlier studies (23).

The gender-based analysis of mandibular anterior teeth revealed nuanced differences in root canal configurations. Mandibular central incisors demonstrated a significantly higher prevalence of the ^1^TN^1^ configuration in females (69.89%) compared to males (64.99%), with statistically significant variations. The findings of our study align with those of Alkahtany et al. (51) in a Saudi population, which demonstrated a statistically significant difference in mandibular incisors among men compared to women. Furthermore, in a separate study conducted on a Saudi population, Mohamed et al. (52) discovered that women exhibited a notably greater incidence of two root canals compared to men. This gender-related variation is also consistent with the study conducted by Baybars and Yelers (53), who also found significant differences in root canal morphology between genders in a Turkish population. Similarly, significant gender-related differences were observed in mandibular lateral incisors, with females exhibiting a higher prevalence of the ^1^TN^1^ configuration (69.04%) compared to males (61.04%). These findings are supported by the study by Verma et al. (54), which highlighted gender-based variations in root canal morphology in Indian populations. Conversely, among Caucasian (6) and Chinese groups (55), men had a greater likelihood of possessing more than one canal compared to women. The observed discrepancies may be attributed to variances in sample sizes and ethnic backgrounds. In contrast, no significant gender differences were found in the root canal configurations for mandibular canines, suggesting a consistent distribution of root canal patterns across genders in this tooth type. This is in line with the research by Qing-ping and Xing (56), which reported similar findings in the Chinese population.

The statistically significant age-related variations observed in selected tooth groups are not merely of academic interest but carry important clinical implications. With increasing age, continuous dentin deposition and progressive canal calcification may alter canal dimensions, complexity, and accessibility, thereby influencing endodontic procedures such as access cavity preparation, canal negotiation, and irrigation effectiveness. The present findings, particularly for maxillary lateral incisors and mandibular canines, suggest that clinicians should anticipate greater anatomical variability and potential treatment challenges in specific age groups. Recognizing these patterns can improve diagnostic accuracy, reduce the risk of missed canals, and contribute to more predictable treatment outcomes.

The classification system of root canal morphology by Ahmed et al. (21) used in this study has provided an advanced framework for categorizing the complex variations found in maxillary and mandibular anterior teeth. This comprehensive system included detailed criteria for identifying different canal types and enhancing precision and consistency in classification. By integrating both traditional and novel configurations, Ahmed et al.'s classification improves our understanding of root canal morphology. This system underscores the importance of meticulous evaluation of root canal morphology, contributing to the detailed and systematic categorization evident in our study.

## Limitations

This study has some limitations. Firstly, the sample size was limited to a specific demographic, potentially limiting generalizability to broader populations. Methodological variations in CBCT imaging protocols and operator interpretations could introduce biases, highlighting the need for standardized procedures. Additionally, the cross-sectional design provides a static view of root canal configurations, warranting future studies to explore changes over time and in response to treatments. An additional limitation of this study is the use of CBCT data acquired from two different imaging systems with varying acquisition parameters. Although such variability may theoretically influence image quality and canal detectability, this potential effect was minimized through strict examiner calibration, standardized image assessment conditions, and the use of voxel resolutions demonstrated in previous studies to be sufficient for accurate root canal evaluation. However, future studies using fully standardized imaging protocols across centres may further strengthen comparative analyses.

Although multivariate modeling could theoretically offer additional insights, the large number of rare canal configuration categories in the present dataset would limit model stability and interpretability. Consequently, chi-square analysis was adopted to provide robust and clinically interpretable results aligned with the descriptive and exploratory objectives of the study.

## Conclusion

The study successfully identified the predominant root canal configurations in a Pakistani subpopulation, revealing significant age and gender-related differences. The predominance of the single root with single (^1^TN^1^) configuration underscores its clinical relevance and prevalence across different demographic factors. The findings emphasize the importance of demographic considerations in endodontic treatment planning, contributing valuable regional data to the field. Ahmed et al.'s classification system enhanced the systematic categorization of root canal configurations, contributing to the comprehensive understanding presented in this study.

## Data Availability

The raw data supporting the conclusions of this article will be made available by the authors, without undue reservation.
